# Differences in Demographic and Clinical Characteristics of Patients With Depressive vs. Manic First Episode of Bipolar Disorder

**DOI:** 10.3389/fpsyt.2021.616415

**Published:** 2021-02-04

**Authors:** Zhonggang Wang, Yuying Cao, Yaya Zhu, Kunkun Li, Xianfei Jiang, Chuanjun Zhuo, Patrick Triplett, Jie Li

**Affiliations:** ^1^Institute of Mental Health, Tianjin Anding Hospital, Tianjin Medical University, Tianjin, China; ^2^Department of Psychiatry, Jining Psychiatric Hospital, Jining, China; ^3^Department of Medical Imaging, Affiliated Hospital of Jining Medical University, Jining, China; ^4^Department of Psychiatry, Affiliated Xuzhou Oriental Hospital of Xuzhou Medical University, Xuzhou, China; ^5^Department of Psychiatry and Behavioral Sciences, Johns Hopkins School of Medicine, Baltimore, MD, United States

**Keywords:** bipolar disorder, clinical characteristics, first onset symptoms, follow-up visit, recurrence rates

## Abstract

**Background:** Bipolar disorder is a serious mental disease marked by episodes of depression, mania, hypomania, or mixed states. Patients with bipolar disorder may present with different symptoms at first onset. The aim of this study is to compare demographic and clinical variables based on a patient's first episode of bipolar disorder, including risk of recurrence over a 2-year period.

**Methods:** A large cohort (*N* = 742) of patients with bipolar disorder in China was analyzed. Patients were divided into two groups according to their first episode of bipolar disorder, either depression or mania. Patients in mixed state first episode were classified based on predominant symptoms. Three hundred eighteen patients of the cohort had a first episode of mania and 424 patients had initial symptoms of depression. Demographic and clinical data were collected. All patients were followed up for 24 months. Data on compliance with follow-up appointments and recurrence of symptoms after 6, 12, 18, and 24 months were collected. Clinical characteristics (course of disease, age of onset, psychiatric family history, etc.) were compared between the mania group and depression groups.

**Results:** More patients with bipolar disorder had a first episode of depression than mania (57.14 vs. 42.86%). Compared with the depression group, the mania group had later age of diagnosis of bipolar disorder [(38.64 ± 13.50) vs. (36.34 ± 14.94), *P* = 0.028], lower education level [(9.37 ± 4.34) vs. (10.17 ± 4.81), *P* = 0.017] and longer latency between an initial episode of psychiatric symptoms and formal bipolar diagnosis [(10.80 ± 10.76) vs. (8.85 ± 9.90), *P* = 0.012]. More patients in the mania group were male and without psychotic symptoms (all *P* < 0.05). In comparison with the mania group, more patients in the depression group were female, with higher frequency of a reported precipitating event before first mood episode (all *P* < 0.05). Compared with the depression group, the mania group had more recurrences of illness at the end of 12 months (*Z* =-2.156, *P* = 0.031), 18 months (*Z* =-2.192, *P* = 0.028), and 24 months (*Z* = −2.364, *P* = 0.018).

**Conclusions:** In our study, there are a number of differences in demographic and clinical characteristics of patients with different onset syndromes of bipolar disorder. These differences include gender, education level, diagnosis age, the rate of recurrences, and others. These data of a cohort of Chinese patients add to the growing international literature on the relationship between index episode of bipolar disorder and clinical variables and outcomes. These results and further study may allow clinicians to offer patients and families more reliable prognostic information at the onset of disease.

## Introduction

Bipolar disorder is a chronic, recurring psychiatric disorder characterized by episodic shifts in mood, energy, social functioning, and activity levels (1). Bipolar disorder is a leading cause of disability and is associated with a substantial economic burden on society (2–4). It may take between 8 and 10 years from the first onset of symptoms to establish a definitive diagnosis (5). The first episode of bipolar disorder can be a manic or hypomanic symptom cluster, mixed state, or a syndrome of depressive symptoms. There are emerging data on how onset-symptom differences may affect the treatment and prognosis of psychiatric disorders (6). According to previous studies and clinical practice (7, 8), it has been found that there are significant differences in treatment effects and disease prognosis among patients with different onset-symptom types of disease. For example, Colom et al. (9) found most patients with bipolar disorder were classified as depressive polarity (depressive vs. manic, 60.3 vs. 39.7%). Depressive polarity was strongly associated with depressive onset of bipolar disorder and patients with this subtype had a higher mean number of suicide attempts.

The number of studies of the clinical characteristics of bipolar disorder based on onset symptoms (2, 10) is somewhat limited and, to our knowledge, no studies of similar scope and size have been conducted on a Chinese patient population. Further, results of existing studies looking at clinical factors linked to bipolar disorder have had discrepant findings. For example, Tondo et al. found depressive episodes were much longer in duration than manias, but episode duration did not differ among bipolar diagnostic types (11). Rangappa et al. found that the predominant polarity was manic (79%). The onset polarity determined the predominant polarity during the course of illness and this had significant implications for treatment (12). However, Baldessarini (same site as Tondo) found the predominant polarity of bipolar disorder was depressive and patients with first onset depressive symptoms had worse prognoses (2). Other studies have shown bipolar disorder may occur in conjunction with a number of other disorders, such as substance abuse and anxiety disorders (13). Based on the existing literature, we hypothesized that there are clinical and prognostic differences based on onset-type for patients with bipolar disorder. We compared the demographic and clinical characteristics of Chinese patients with bipolar disorder with different first onset symptoms and their recurrence status during follow up visits over 2 years. The aim of this study is to provide an objective basis for evaluating clinical and demographic characteristics of patients with bipolar disorder, which may provide useful information on disease prognosis and other outcomes.

## Participants and Methods

### Study Design and Participants

Patients included in this study were a convenience sample of all inpatients with bipolar disorder admitted to Jining Psychiatric Hospital from January 1st, 2016 to December 31th 2016. There were 372 males [Duration of illness (9.75 ± 10.60) years] and 370 females [Duration of illness (9.61 ± 10.04) years] aged 18–60 years old, with a current diagnosis of bipolar disorder recruited for this study. Selection criteria included: a primary diagnosis of bipolar disorder according to the International Classification of Diseases-10 (ICD-10), a score of 5 or lower on the Bech-Rafaelsen mania rating scale (BRMS) (14), a score of 8 or lower on the Hamilton depression scale (HAMD) (15), the ability to understand and sign the consent form, and no substance use disorder in the last 3 months. Subjects were divided into two subgroups according to their onset symptoms. The mania group included 318 patients; the other 424 patients belonged to the depression group. This study was carried out in accordance with the principles of informed consent and the Institutional Review Board for Jining Psychiatric Hospital approved the study and its informed consent procedures. Subjects provided written, informed consent after receiving a complete description of the study and after being given chances to ask questions about their participation.

### Assessments of Mental Condition

All participants were evaluated for ICD-10 bipolar disorders using the MINI- International Neuropsychiatric Interview (MINI). Final diagnoses were made by two independent clinicians. The nature of the first episode of bipolar disorder was derived from patient history in the medical record, patient self-report, or family report. The first episode was often described in the medical record, though we would consult the patient or their relatives to better identify the first episode type if it was ambiguous in the chart. The general demographic data and disease clinical data were collected by questionnaire. In order to determine the state of disease including psychotic symptoms, all participants were measured by Bech-Rafaelsen mania rating scale (BRMS), the Hamilton depression scale 24-item version (HAMD-24) and the Positive and Negative Syndrome Scale (PANSS). These scores for a patient's current mental state were obtained primarily to establish that they met the study's selection criteria.

#### Bech-Rafaelsen Mania Rating Scale (BRMS) (14)

The BRMS was used to assess the severity of manic symptoms. A higher score correlates with greater symptom severity. The scores between 0 and 4 indicate no obvious manic symptoms, scores from 6 to 10 are mild manic symptoms, and scores ≥22 would be considered severe manic symptoms.

#### Hamilton Depression Scale-24 Item Version (HAM-D-24) (15)

The HAM-D was used to assess the severity of depressive symptoms. Severe depression was marked by scores ≥35. The scores of mild or moderate depression were ≥20 but <35. Any patients with scores ≤ 7 were considered to have no depressive symptoms.

#### The Positive and Negative Syndrome Scale (PANSS) (16, 17)

The PANSS is composed of seven items on the positive symptom scale, seven items of negative symptom scale and 16 items of general psychopathology. In this study, the positive symptom scale was used to assess whether the patients had psychotic symptoms and if so, these psychotic symptoms' severity. Data gathered from this assessment are applied to the PANSS ratings. Each of the 30 items is accompanied by a specific definition as well as detailed anchoring criteria for all seven rating points. These seven points represent increasing levels of psychopathology: 1-absent, 2-minimal, 3-mild, 4-moderate, 5-moderate severe, 6-severe, and 7-extreme.

### Analysis Variables

Demographic variables and clinical variables were collected and analyzed in this study. Demographic variables included: age (years), education (years), sex, address (urban/rural), and marriage (%). Clinical variables included: latency between first psychiatric symptoms and bipolar diagnosis (years), first psychiatric symptom onset age (years), inpatient length of stay (first admission), age at confirmed bipolar diagnosis (years), family psychiatric history, suspected precipitating event for first episode (yes/no), psychotic symptoms (yes/no), average return visit time (months), the number of patients seen at return visit (12, 18, 24 months), relapse episodes during 6 months intervals (12, 18, 24 months).

### Statistical Analysis

All statistical analyses were performed using IBM SPSS version 20.0 (Statistical Package for the Social Sciences). Descriptive statistics and frequency distributions were calculated for demographic and illness history variables (e.g., sex, race, living address, age, age of onset of first depressive, and manic symptoms). Between-group demographic and clinical characteristics were compared using parametric and non-parametric methods, as appropriate. Two independent samples *t*-test (two-sided) were performed to compare the variables obeying normal distribution within two groups. Independent sample Rank- Sum test was used to compare non-normal distribution variables. The classification variables were expressed by the number of cases and the proportion of components (*n*, %), and the differences between groups were compared by chi-square test. The significance level was set at 0.05 using bilateral test.

## Results

### Participant Characteristics

There were 742 participants in the study [318 (42.86%) patients with first episode depression and 424 (57.14%) patients with first episode mania]. We compared the demographic characteristics and clinical characteristics between the two groups (Table 1).

**Table 1:** Comparison of demographic characteristics between bipolar patients with different first-episode symptoms [$\bar{X}$ ± S or N (%)].

		**Mania patients group *N* = 318 (42.86%)**	**Depression patients group *N* = 424 (57.14%)**	**Statistics**	
				***t* or χ2**	***P*-values**
Age (years, mean ± SD)		38.64 ± 13.50	36.34 ± 14.94	2.197	=0.028^*^
Education (years, mean ± SD)		9.37 ± 4.34	10.17 ± 4.81	−2.391	=0.017^*^
Sex (%)	Male	179 (56.29)	193 (45.52)	8.432	=0.004^*^
	Female	139 (43.71)	231 (54.48)		
Address (%)	Urban	50 (15.72)	109 (25.70)	10.759	=0.001^*^
	Rural	268 (84.28)	315 (74.29)		
Marriage (%)	Yes	232 (72.96)	282 (66.51)	5.513	=0.063
	No	72 (22.64)	128 (30.19)		
	Divorce	14 (4.40)	14 (3.30)		

* *Significant differences with p < 0.05 were indicated in bold*.

### Demographic Characteristics Between the Mania Group and the Depression Group

Compared to the depression group, the mania group was older (*t* = 2.197, *P* = 0.028) and had less education (*t* = −2.391, *P* = 0.017). In addition, more patients were male in the mania group. More patients were female in the depression group; the sex composition ratio of two groups was significantly different (χ^2^ = 8.432, *P* = 0.004). A large ratio of patients were from rural areas in both the depression group and the mania group, however, the rural/urban composition ratio of the two groups was significantly different (χ*2* = 10.759, *P* = 0.001). There was no significant difference in the ratios of marital status between the two groups (χ^2^ = 5.513, *P* = 0.063), most patients were married in the two groups and the percentage of married patients were 72.96 vs. 66.51% in the depression group and the mania group, respectively (Table 1).

### Clinical Characteristics Between the Mania Group and the Depression Group

The mania group had significantly longer latency between an initial episode of psychiatric symptoms and getting a definitive diagnosis of bipolar disorder than the depression group [(10.80 ± 10.76) vs. (8.85 ± 9.90), *t* = 2.525, *P* = 0.012]. In comparison with the mania group, the depression group patients were younger when they were diagnosed [(38.64 ± 13.50) vs. (36.34 ± 14.94), *t* = 2.197, *P* = 0.028]. In addition, some clinical variables were distinct between the two groups, including the proportion of patients whom clinicians believed had a triggering life event to their mood episode (χ^2^ = 10.649, *P* = 0.001), as well as the proportions of patients with psychotic symptoms (χ^2^ = 5.577, *P* = 0.018). The rate of patients with significant life events in the depression group was higher than for the mania group (34.67 vs. 23.58%, χ^2^ = 10.649, *P* = 0.001). The rate of psychotic symptoms in the depression group was also higher than the mania group (45.75 vs. 37.53%, χ^2^ = 5.577, *P* = 0.018). There were no significant differences between the two groups in first onset age [(27.84 ± 10.79) vs. (27.49 ± 11.53), *t* = 0.432, *P* = 0.666] or psychiatric family history (χ^2^ = 1.663, *P* = 0.197) (Table 2).

**Table 2:** Comparison of clinical characteristics between patients with bipolar disorder with different first-episode symptoms [($\bar{X}$ ± S) or N (%)].

		**Mania patients group (*N* = 318)**	**Depression patients group (*N* = 424)**	**Statistics**	
				***t* or **χ**^2^ or Z**	***P*-values**
Latency between first psychiatric symptoms and bipolar diagnosis (years, mean ± SD)		10.80 ± 10.76	8.85 ± 9.90	2.525	=0.012^*^
First psychiatric symptom onset age (years, mean ± SD)		27.84 ± 10.49	27.49 ± 1 1.53	0.432	=0.666
Length of stay (days, mean ± SD)		42.742 ± 33.64	42.828 ± 33.78	−0.034	=0.973
Confirmed bipolar diagnosis age (years, mean ± SD)		38.64 ± 13.50	36.34 ± 14.94	2.197	=0.028
Family history	Yes	78 (32.50)	122 (28.77)	1.663	=0.197
	No	240 (75.50)	302 (71.23)		
Suspected precipitating event for first episode	Yes	75 (23.58)	147 (34.67)	10.649	=0.001^*^
	No	243 (76.42)	277 (65.33)		
Psychotic symptoms	Yes	118 (37.52)	194 (45.75)	5.577	=0.018^*^
	No	200 (62.89)	230 (54.25)		

* *Significant differences with p < 0.05 were indicated in bold*.

### Number of Recurrences Between the Mania Group and the Depression Group During 24 Months Follow-Up

There were significant differences between two groups in the number of recurrences at the end of 12 months (*Z* = −2.156, *P* = 0.031), 18 months (*Z* = −2.192, *P* = 0.028), and 24 months (*Z* = −2.364, *P* = 0.018) (Table 3).

**Table 3:** The rate of regular return visit between the mania group and the depression group during 24 months follow-up [(X ± S) or N (%)].

	**Mania patients group (*N* = 318)**	**Depression patients group (*N* = 424)**	***t* or *Z***	***P*-values**
Average return visit time (months)	13.25 ± 11.99	14.34 ± 11.79	−1.240	0.215
Patients seen at return visit ≥6 months	187 (58.81)	275 (64.86)	–	–
Relapse episodes during 6 months	0.10 ± 0.32	0.15 ± 0.37	−0.462	0.644
Patients seen at return visit ≥12 months	166 (52.20)	226 (53.30)	–	–
Relapse episodes during 12 months	0.36 ± 0.64	0.25 ± 0.51	−2.156	0.031^*^
Patients seen at return visit ≥18 months	138 (43.40)	191 (45.05)	–	–
Relapse episodes during 18 months	0.48 ± 0.80	0.34 ± 0.60	−2.192	0.028^*^
Patients seen at return visit ≥24 months	92 (28.93)	131 (30.90)	–	–
Relapse episodes during 24 months	0.60 ± 0.89	0.42 ± 0.68	−2.364	0.018^*^

* *Significant differences with p < 0.05 were indicated in bold*.

## Discussion

Bipolar disorder is a chronic, relapsing and remitting disease characterized by recurrent episodes of manic, hypomanic, depressive or mixed symptoms. Bipolar disorder can have a lifelong impact on the overall health status, functioning, and quality of life of patients. Onset of bipolar disorder occurs usually in adolescence or in early adulthood, and its clinical manifestations are variable (18, 19). A number of prior studies have examined two major onset types—first episode of mania/hypomania or depression and some researchers (2, 20) have found different characteristics between patients within two groups. Distinctions between the two onset types include the course of illness, age of onset and symptoms of psychosis, in addition to others. Studies (2, 21) suggest that exploring the characteristics of bipolar disorder may be helpful in better evaluating any disease-related changes and to guide clinical diagnosis and treatment. Baldessarini et al. (22) followed SCID-based, DSM-IV diagnosed bipolar I patients (*n* = 303) systematically and prospectively for 2 years to estimate the percentage of weeks in specific morbid states from first lifetime major episodes and found morbidity was strongly predicted by first-episode polarity. In this study, we examine the clinical features of patients with different initial symptoms of bipolar disorder and outcomes related to their condition after diagnosis.

This study found the first onset syndrome of 57.14% of patients with bipolar disorder were depressive, consistent with some prior studies. Besnier et al. (23) found the rate of depressive first episode of bipolar disorder was higher than the rate of manic first episodes. Besnier et al. (23) further noted that patients with first episode depression had the following characteristics: longer disease course, more frequent recurrence times, higher rate of depression recurrence, and higher incidence of suicidal behavior, when compared with patients whose first episode was mania. Through the study of 1,081 adult patients with bipolar disorder, Baldessarini et al. (2) also found depressive first episodes were most common in bipolar disorder, with a ranking of onset symptoms of depression (59%) > mania (13%) > psychosis (8.0%) > anxiety (7.6%) > hypomania (6.7%) > mixed states (5.5%). Carvalho et al. (24) found patients with first-onset depressive symptoms had worse overall prognosis compared to patients with first-onset manic/hypomanic symptoms.

A commonly noted feature of the study of bipolar disorder is delay in diagnosis. Fritz et al. (5) tried to study predictors of diagnosis change from depression to bipolar disorder in order to understand patient features and explain this delay. Their study included ninety patients who were initially diagnosed with depression and later diagnosed with bipolar disorder. They found the mean delay in diagnostic conversion was 8.74 years (8.74 ± 9.40 years). Our study similarly found an average delay to bipolar diagnosis for patients with an initial manic episode of 10.8 years (10.8 ± 10.76 years), and 8.85 years (8.85 ± 9.90 years) delay in diagnosis for patients with an index depressive episode. The reasons for this latency in establishing a diagnosis is likely determined by a number of factors and merits further attention, as earlier diagnosis may provide patients with needed treatments earlier and may possibly lead to better outcomes.

In our analysis, age of onset of psychiatric symptoms was similar for both groups [mania (27.84 ± 10.79) years and depression (27.49 ± 11.53) years]. These findings are consistent with several previous reports (25–27). Prior studies (28, 29) have shown that the educational level of patients with bipolar disorder has an influence on their adherence to treatment. Patients with higher levels of education tend to have better treatment adherence than patients with lower level of education. Our study similarly found that patients with greater education, particularly those with first onset depression, had a better rate of follow up adherence. This result may be related in part to better compliance with treatment in the patients with the first episode of depression.

We found the rate of patients with significant life events in the depression group was higher than for the mania group (34.67 vs. 23.58%). This point suggested that patients with bipolar disorder who have some form of traumatic or otherwise powerful life event near the onset of their index episode are primarily depressed. A prior study (30) found a similar connection. Azorin et al. further found depressive first onset patients with bipolar disorder had more depressive temperaments, a first episode triggered by stress, an illness course with more episodes, more suicide attempts and greater residual symptoms.

We also found the rate of psychotic symptoms in the depression group was higher than the mania group, similar to a recent study (31). This study found many patients with bipolar disorder had prominent psychotic symptoms as a feature of a depressive episode. Psychotic symptoms may occur during a manic episode as well and psychotic depression may evolve to psychotic mania. Studies have found significant clinical correlations for patients with bipolar disorder syndromes with psychotic features, such as early age of onset, comorbid personality disorder, number of hospitalizations, and suicidality (32). This underscores the importance of early and effective treatment of psychotic symptoms in patients with bipolar disorder.

Compared with the depression group, the mania group had more recurrences of illness at the end of 12 months [(0.36 ± 0.64) vs. (0.25 ± 0.51), *p* = 0.031], 18 months [(0.48 ± 0.80) vs. (0.34 ± 0.60), *p* = 0.028], and 24 months [(0.60 ± 0.89) vs. (0.42 ± 0.676), *p* = 0.018]. This seems to suggest that the different onset types can affect the rate of recurrence, the number of mood episodes, or even the rate or the degree of recovery. Studies (33, 34) have reported that longer illness duration predicted a longer time to recovery, and that the number of previous mood episodes and illness duration are associated with the likelihood and speed of recovery among patients with bipolar disorder. Baldessarini et al. (22) followed 247 patients with different initial major episodes of bipolar disorder prospectively for 24 months, and found more frequent manias, hypomanias, and perhaps more psychosis during follow-up after initial mania than other subtypes of bipolar disorder.

## Conclusion

In conclusion, there are a number of differences in demographic and clinical characteristics of patients with different onset syndromes of bipolar disorder. Patients with bipolar disorder were more likely to present with an index episode of depression and these patients were more often female and had some temporally-related life event associated with the symptoms. Patients with an index episode of mania were more likely to be male, less educated, had greater latency between initial symptoms and diagnosis, were older when diagnosed, and were more likely to have recurrences noted at follow-up. Further understanding of these differences may be helpful for providers in estimating outcomes and prognosis for patients with bipolar disorder, with implications for treatment choices as well as future directions for research. In future studies, we plan to extend the follow-up period in order to provide more longitudinal data in hopes of further exploring the differences in clinical characteristics and prognosis in patients with bipolar disorder based on first episode found in this study.

### Limitations

The study was conducted entirely in one area of China, which may affect generalizability of findings. The study also only covered a 2 year span looking at recurrences and adherence with follow-up appointments and thus may not convey a full picture on the life course of the condition. Another shortcoming of our approach is that hypomania as a presenting syndrome is often undetected or under-detected. In future study, we may consider using a structured or semi-structured interview with broader scope (such as the SCID-5) to better capture this subset of patients with bipolar disorder. A more structured interview may also help more reliably detect mixed states than we were able to for this study.

## Data Availability Statement

The raw data supporting the conclusions of this article will be made available by the authors, without undue reservation.

## Ethics Statement

The studies involving human participants were reviewed and approved by Jining Psychiatric Hospital. The patients/participants provided their written informed consent to participate in this study.

## Author Contributions

ZW: conceptualization, formal analysis, writing—original draft, and supervision. YC: data curation and writing—original draft. YZ: formal analysis and data curation. KL: formal analysis and writing—original draft. XJ: data curation. CZ: conceptualization. PT: conceptualization and writing—original draft. JL: conceptualization. All authors contributed to the article and approved the submitted version.

## Conflict of Interest

The authors declare that the research was conducted in the absence of any commercial or financial relationships that could be construed as a potential conflict of interest.
